# Contingency Table Analysis and Inference via Double Index Measures

**DOI:** 10.3390/e24040477

**Published:** 2022-03-29

**Authors:** Christos Meselidis, Alex Karagrigoriou

**Affiliations:** Laboratory of Statistics and Data Analysis, Department of Statistics and Actuarial-Financial Mathematics, University of the Aegean, Karlovasi, GR-83200 Samos, Greece; alex.karagrigoriou@aegean.gr

**Keywords:** double index divergence test statistic, multivariate data analysis, conditional independence, cross tabulations

## Abstract

In this work, we focus on a general family of measures of divergence for estimation and testing with emphasis on conditional independence in cross tabulations. For this purpose, a restricted minimum divergence estimator is used for the estimation of parameters under constraints and a new double index (dual) divergence test statistic is introduced and thoroughly examined. The associated asymptotic theory is provided and the advantages and practical implications are explored via simulation studies.

## 1. Introduction

The concept of distance or divergence is known since at least the time of Pearson, who, in 1900, considered the classical goodness-of-fit (gof) problem by considering the distance between observed and expected frequencies. The problem for both discrete and discretized continuous distributions have been in the center of attention for the last 100+ years. The classical set-up is the one considered by Pearson where a hypothesized *m*-dimensional multinomial distribution, say $Multi(N,p_{1},\ldots ,p_{m})$ is examined as being the underlying distributional mechanism for producing a given sample of size *N*. The problem can be extended to examine the homogeneity (in terms of the distributional mechanisms) among two independent samples or the independence among two population characteristics. In all such problems we are dealing with cross tabulations or crosstabs (or contingency tables). Problems of such nature appear frequently in a great variety of fields including biosciences, socio-economic and political sciences, actuarial science, finance, business, accounting, and marketing. The need to establish for instance, whether the mechanisms producing two phenomena are the same or not is vital for altering economic policies, preventing socio-economic crises or enforcing the same economic or financial decisions to groups with similar underlying mechanisms (e.g., retaining the insurance premium in case of similarity or having different premiums in case of diversity). It is important to note that divergence measures play a pivotal role also in statistical inference in continuous settings. Indeed, for example, in [1] the authors investigate the multivariate normal case while in a recent work [2], the modified skew-normal-Cauchy (MSNC) distribution is considered, against normality.

Let us consider the general case of two *m*-dimensional multinomial distributions for which each probability depends on an *s*-dimensional unknown parameter, say θ$={\left(\theta_{1},\ldots ,\theta_{s}\right)}^{⊤}$. A general family of measures introduced by [3] is the $d_{\Phi }^{\alpha }$ family defined by

(1) $$d_{\Phi }^{\alpha }\left(p(\theta ),q(\theta )\right)=\sum_{i=1}^{m}q_{i}{\left(\theta \right)}^{1+\alpha }\Phi (\frac{p_{i}\left(\theta \right)}{q_{i}\left(\theta \right)});\;\alpha >0,\;\Phi \in F^{*}$$

where α is a positive indicator (index) value, $p(\theta )={\left(p_{1}\left(\theta \right),\ldots ,p_{m}\left(\theta \right)\right)}^{⊤}$ and $q(\theta )=(q_{1}\left(\theta \right),$ …, $q_{m}{\left(\theta \right))}^{⊤}$, $F^{*}$ is a class of functions s.t. $F^{*}=\{\Phi \left(\cdot \right):\Phi \left(x\right)$ strictly convex, $x\in R^{+},$$\Phi \left(1\right)=\Phi^{\prime }\left(1\right)=0,$$\Phi^{\prime \prime }\left(1\right)\neq 0$ and by convention, Φ(0/0)=0 and 0Φ(p/0) = $\lim_{x\to \infty }\left[\Phi \left(x\right)/x\right]\}$.

Note that the well known Csiszar family of measures [4] is obtained for the special case where the indicator is taken to be equal to 0 while the classical Kullback–Leibler (KL) distance [5] is obtained if the indicator α is equal to 0 and at the same time the function Φ(·) is taken to be $\Phi \left(x\right)\equiv \Phi_{KL}\left(x\right)=$xlog(x)orxlog(x)−x+1.

The function

$$\Phi_{\lambda }\left(x\right)=\frac{1}{\lambda (\lambda +1)}\left[x\left(x^{\lambda }-1\right)-\lambda \left(x-1\right)\right]\in F^{*},\lambda \neq 0,-1$$

is associated with the Freeman–Tukey test when λ=−1/2, with the recommended Cressie and Read (CR) power divergence [6] when λ=2/3, with the Pearson’s chi-squared divergence [7] when λ=1 and with the classical KL distance when λ→0.

Finally, the function

$$\Phi_{\alpha }\left(x\right)\equiv \left(\lambda +1\right)\Phi_{\lambda }{\left(x\right)|}_{\lambda =\alpha }=\frac{1}{\alpha }\left[x\left(x^{\alpha }-1\right)-\alpha \left(x-1\right)\right],\;\alpha \neq 0$$

produces the BHHJ or $\Phi_{\alpha }$-power divergence [8] given by

$$d_{\Phi_{\alpha }}^{\alpha }\left(p(\theta ),q(\theta )\right)=\sum_{i=1}^{m}q_{i}^{\alpha }\left(\theta \right)\left\{q_{i}\left(\theta \right)-p_{i}\left(\theta \right)\right\}+\frac{1}{\alpha }\sum_{i=1}^{m}p_{i}\left(\theta \right)\left\{p_{i}^{\alpha }\left(\theta \right)-q_{i}^{\alpha }\left(\theta \right)\right\}.$$

Assume that the underlying true distribution of an *m*-dimensional multinomial random variable with *N* experiments, is

$$X={\left(X_{1},\ldots ,X_{m}\right)}^{⊤}∼Multi\left(N,p={\left(p_{1},\ldots ,p_{m}\right)}^{⊤}\right)$$

where p is, in general, unknown, belonging to the parametric family

(2) $$P=\left\{p\left(\theta \right)={\left(p_{1}\left(\theta \right),\ldots ,p_{m}\left(\theta \right)\right)}^{⊤}:\theta ={\left(\theta_{1},\ldots ,\theta_{s}\right)}^{⊤}\in \Theta \subset R^{s}\right\}.$$

The sample estimate $\hat{p}={\left({\hat{p}}_{1},\ldots ,{\hat{p}}_{m}\right)}^{⊤}$ of p is easily obtained by ${\hat{p}}_{i}=x_{i}/N$ where $x_{i}$ is the observed frequency for the *i*-th category (or class).

Divergence measures can be used for estimating purposes by minimizing the associated measure. The classical estimating technique is the one where (1) we take α=0 and $\Phi \left(x\right)=\Phi_{KL}\left(x\right)$. Then, the resulting KL minimization is equivalent to the classical maximization of the likelihood producing the well-known Maximum Likelihood Estimator (MLE, see ([9], Section 5.2)). In general, the minimization with respect to the parameter of interest of the divergence measure, gives rise to the corresponding minimum divergence estimator (see, e.g., [6,10,11]). For the case where constraints are involved the case associated with Csiszar’s family of measures was recently investigated [12]. For further references, please refer to [13,14,15,16,17,18,19,20,21].

Consider the hypothesis

(3) $$\begin{array}{c} H_{0}:p=p\left(\theta_{0}\right)\;vs.\;H_{1}:p\neq p\left(\theta_{0}\right),\;\theta_{0}={\left(\theta_{01},\ldots ,\theta_{0s}\right)}^{⊤}\in \Theta \subset R^{s} \end{array}$$

where p is the vector of the true but unknown probabilities of the underlying distribution and $p(\theta_{0})$ the vector of the corresponding probabilities of the hypothesized distribution which is unknown and falls within the family of P with the unknown parameters satisfying in general, certain constraints, e.g., of the form c(θ)=0, under which the estimation of the parameter will be performed. The purpose of this work is twofold: having as a reference the divergence measure given in (1), we will first propose a general double index divergence class of measures and make inference regarding the parameter estimators involved. Then, we proceed with the hypothesis problem with the emphasis given to the concept of conditional independence. The innovative idea proposed in this work is the duality in choosing among the members of the general class of divergences, one for estimating and one for testing purposes which may not be necessarily, the same. In that sense, we propose a double index divergence test statistic offering the greatest possible range of options, both for the strictly convex function Φ and the indicator value α>0.

Thus, the estimation problem can be examined considering expression (1) using a function $\Phi_{2}\in F^{*}$ and an indicator $\alpha_{2}>0$:

(4) $$d_{\Phi_{2}}^{\alpha_{2}}\left(p,p(\theta )\right)=\sum_{i=1}^{m}p_{i}^{1+\alpha_{2}}\left(\theta \right)\Phi_{2}\left(\frac{p_{i}}{p_{i}\left(\theta \right)}\right)$$

the minimization of which with respect to the unknown parameter, will produce the restricted minimum $\left(\Phi_{2},\alpha_{2}\right)$ divergence (rMD) estimator

(5) $${\hat{\theta }}_{\left(\Phi_{2},\alpha_{2}\right)}^{r}=\arg\underset{\theta \in \Theta :c(\theta )=0}{\inf}d_{\Phi_{2}}^{\alpha_{2}}\left(\hat{p},p\left(\theta \right)\right)$$

for some constraints c(θ)=0. Observe that the unknown vector of underlying probabilities has been replaced by the vector of the corresponding sample frequencies $\hat{p}$. Then, the testing problem will be based on

(6) $$d_{\Phi_{1}}^{\alpha_{1}}\left(\hat{p},p({\hat{\theta }}_{\left(\Phi_{2},\alpha_{2}\right)}^{r})\right)=\sum_{i=1}^{m}p_{i}^{1+\alpha_{1}}\left({\hat{\theta }}_{\left(\Phi_{2},\alpha_{2}\right)}^{r}\right)\Phi_{1}\left(\frac{{\hat{p}}_{i}}{p_{i}\left({\hat{\theta }}_{\left(\Phi_{2},\alpha_{2}\right)}^{r}\right)}\right)$$

where $\Phi_{1}\left(\cdot \right)$ and $\alpha_{1}$ may be different from the corresponding quantities used for the estimation problem in (4). Finally, the duality of the proposed methodology surfaces when the testing problem is explored via the dual divergence test statistic formulated on the basis of the double-α-double-Φ divergence given by

(7) $$d_{\Phi_{1}}^{\alpha_{1}}\left(\hat{p},p({\hat{\theta }}_{\left(\Phi_{2},\alpha_{2}\right)}^{r})\right)$$

where $\Phi_{1},\Phi_{2}\in F^{*}$ and $\alpha_{1},\alpha_{2}>0$.

The remaining parts of this work are: Section 2 presents the formal definition and the asymptotic properties of the rMD estimator (rMDE). Section 3 deals with the general testing problem with the use of rMDE. The associated set up for the case of three-way contingency tables is developed in Section 4 with a simulation section emphasizing on the conditional independence of three random variables. We close this work with some conclusions.

## 2. Restricted Minimum (Φ,α)-Power Divergence Estimator

In what follows, we will provide the formal definition and the expansion of the rMD estimator and prove its asymptotic normality. The assumptions required for establishing the results of this section for the rMD estimator under constraints, are provided below:

**Assumption** **1.**

$\left(A_{0}\right)$

*$\;f_{1}\left(\theta \right),\ldots ,f_{\nu }\left(\theta \right)$ are the constrained functions on the s-dimensional parameter θ, $f_{k}\left(\theta \right)=0$, k=1,…,ν and ν<s<m−1;*

$\left(A_{1}\right)$

 *There exists a value $\theta_{0}\in \Theta$, such that $X={\left(X_{1},\ldots ,X_{m}\right)}^{⊤}∼Multi\left(N,p(\theta_{0})\right)$;*

$\left(A_{2}\right)$

 *Each constraint function $f_{k}\left(\theta \right)$ has continuous second partial derivatives;*

$\left(A_{3}\right)$

 *The ν×s and m×s matrices*
  $$Q\left(\theta_{0}\right)={\left(\frac{\partial f_{k}\left(\theta_{0}\right)}{\partial \theta_{j}}\right)}_{\begin{array}{c} k=1,\ldots ,\nu \\ j=1,\ldots ,s \end{array}}\;\;\begin{array}{c} \mathrm{and} \end{array}\;\;J\left(\theta_{0}\right)={\left(\frac{\partial p_{i}\left(\theta_{0}\right)}{\partial \theta_{j}}\right)}_{\begin{array}{c} i=1,\ldots ,m\\ j=1,\ldots ,s \end{array}}$$
*are of full rank;*

$\left(A_{4}\right)$

 ***p*** *(θ) has continuous second partial derivatives in a neighbourhood of $\theta_{0}$;*

$\left(A_{5}\right)$

 *$\theta_{0}$ satisfies the Birch regularity conditions (see Appendix A and [22]).*

**Definition** **1.** *Under assumptions $\begin{array}{c} \left(A_{0}\right) \end{array}$–$\left(A_{3}\right)$ the rMD estimator of $\theta_{0}$ is any vector in Θ, such that*

(8) $${\hat{\theta }}_{\left(\Phi ,\alpha \right)}^{r}=\arg\inf_{\left\{\theta \in \Theta \subset R^{s}:\;f_{k}\left(\theta \right)=0,k=1,\ldots ,\nu \right\}}d_{\Phi }^{\alpha }\left(\hat{p},p\left(\theta \right)\right).$$

In order to derive the decomposition of ${\hat{\theta }}_{\left(\Phi ,\alpha \right)}^{r}$ the Implicit Function Theorem (IFT) is exploited according to which if a function has an invertible derivative at a point then itself is invertible in a neighbourhood of this point but it cannot be expressed in closed form [23].

**Theorem** **1.** *Under Assumptions $\begin{array}{c} \left(A_{0}\right) \end{array}$–$\left(A_{5}\right)$, the rMD estimator of $\theta_{0}$ is such that*

(9) $$\begin{array}{cc} {\hat{\theta }}_{\left(\Phi ,\alpha \right)}^{r}=\; & \theta_{0}+H\left(\theta_{0}\right){\left(B{\left(\theta_{0}\right)}^{⊤}B\left(\theta_{0}\right)\right)}^{-1}B{\left(\theta_{0}\right)}^{⊤}diag\left(p{\left(\theta_{0}\right)}^{\alpha /2}\right)\times \\ & \times diag\left(p{\left(\theta_{0}\right)}^{-1/2}\right)\left(\hat{p}-p\left(\theta_{0}\right)\right)+o\left(∥\hat{p}-p\left(\theta_{0}\right)∥\right) \end{array}$$

*where ${\hat{\theta }}_{\left(\Phi ,\alpha \right)}^{r}$ is unique in a neighbourhood of $\theta_{0}$ and*

$$\begin{array}{ccc} H(\theta_{0})= & \;I-{\left(B{\left(\theta_{0}\right)}^{⊤}B\left(\theta_{0}\right)\right)}^{-1}Q{\left(\theta_{0}\right)}^{⊤}\times & \\ \; & \times {\left(Q\left(\theta_{0}\right){\left(B{\left(\theta_{0}\right)}^{⊤}B\left(\theta_{0}\right)\right)}^{-1}Q{\left(\theta_{0}\right)}^{⊤}\right)}^{-1}Q\left(\theta_{0}\right), \end{array}$$

$$B\left(\theta_{0}\right)=diag\left(p{\left(\theta_{0}\right)}^{\alpha /2}\right)A\left(\theta_{0}\right),\;\mathrm{while}\;A\left(\theta_{0}\right)=diag\left(p{\left(\theta_{0}\right)}^{-1/2}\right)J\left(\theta_{0}\right).$$

**Proof.**  Let *V* be a neighbourhood of $\theta_{0}$ on which $p\left(\cdot \right):\Theta \to P\subset l^{m}$ has continuous second partial derivatives where $l^{m}$ is the interior of the unit cube of dimension *m*. Let

$$F=\left(F_{1},\ldots ,F_{\nu +s}\right):l^{m}\times R^{\nu +s}\to R^{\nu +s}$$

with

$$F_{j}\left(p,\lambda ,\theta \right)=\left\{\begin{array}{cc} f_{j}\left(\theta \right), & j=1,\ldots ,\nu \\ \frac{\partial d_{\Phi }^{\alpha }\left(p,p(\theta )\right)}{\partial \theta_{j-\nu }}+\sum_{k=1}^{\nu }\lambda_{k}\frac{\partial f_{k}\left(\theta \right)}{\partial \theta_{j-\nu }},\; & j=\nu +1,\ldots ,\nu +s. \end{array}\right.$$

where $\left(p,\lambda ,\theta \right)=\left(p_{1},\ldots ,p_{m},\lambda_{1},\ldots ,\lambda_{\nu },\theta_{1},\ldots ,\theta_{s}\right)$ and $\lambda_{k}$, k=1,…,ν are the coefficients of the constraints.

It holds that

$$F_{j}\left(p_{1}\left(\theta_{0}\right),\ldots ,p_{m}\left(\theta_{0}\right),0,\ldots ,0,\theta_{01},\ldots ,\theta_{0s}\right)=0,\;j=1,\ldots ,\nu +s$$

and by denoting $\gamma =\left(\gamma_{1},\ldots ,\gamma_{\nu +s}\right)=\left(\lambda_{1},\ldots ,\lambda_{\nu },\theta_{1},\ldots ,\theta_{s}\right)$, the matrix

$$\frac{\partial F}{\partial \gamma }={\left(\frac{\partial F_{j}}{\partial \gamma_{k}}\right)}_{\begin{array}{c} j=1,\ldots ,\nu +s\\ k=1,\ldots ,\nu +s \end{array}}=\left(\begin{array}{cc} 0_{\nu \times \nu } & Q(\theta_{0})\\ Q{\left(\theta_{0}\right)}^{⊤} & \Phi^{\prime \prime }\left(1\right)B{\left(\theta \right)}^{⊤}B\left(\theta \right) \end{array}\right)$$

is nonsingular at $\left(p,\lambda ,\theta \right)=\left(p\left(\theta_{0}\right),\gamma_{0}\right)=\left(p_{1}\left(\theta_{0}\right),\ldots ,p_{m}\left(\theta_{0}\right),0,\ldots ,0,\theta_{01},\ldots ,\theta_{0s}\right)$ with $\gamma_{0}=\left(0_{\nu },\theta_{0}\right)$.

Using the IFT a neighbourhood *U* of $\left(p\left(\theta_{0}\right),\gamma_{0}\right)$ exists, such that ∂F/∂γ is nonsingular and a unique differentiable function $\gamma^{*}=\left(\lambda^{*},\theta^{*}\right)$$:A\subset l^{m}\to R^{\nu +s}$, such that $p(\theta_{0})\in A$ and $\left\{\left(p,\gamma \right)\in U:F\left(p,\gamma \right)=0\right\}=\{\left(p,\gamma^{*}\left(p\right)\right):p\in A\}$ and $\gamma^{*}\left(p\left(\theta_{0}\right)\right)=\left(\lambda^{*}\left(p\left(\theta_{0}\right)\right),\theta^{*}\left(p\left(\theta_{0}\right)\right)\right)=\gamma_{0}$. By the chain rule and for $p=p(\theta_{0})$ we obtain

$$\frac{\partial F}{\partial p(\theta_{0})}+\frac{\partial F}{\partial \gamma_{0}}\frac{\partial \gamma_{0}}{\partial p(\theta_{0})}=0.$$

Then

$$\frac{\partial \theta_{0}}{\partial p(\theta_{0})}=\left(\begin{array}{c} E(\theta_{0})\\ W(\theta_{0}) \end{array}\right)$$

where

$$\begin{array}{cc} E(\theta_{0})=\; & \Phi^{\prime \prime }\left(1\right){\left(Q\left(\theta_{0}\right){\left(B{\left(\theta_{0}\right)}^{⊤}B\left(\theta_{0}\right)\right)}^{-1}Q{\left(\theta_{0}\right)}^{⊤}\right)}^{-1}\times \\ \; & \times Q\left(\theta_{0}\right){\left(B{\left(\theta_{0}\right)}^{⊤}B\left(\theta_{0}\right)\right)}^{-1}B{\left(\theta_{0}\right)}^{⊤}diag\left(p{\left(\theta_{0}\right)}^{\alpha /2}\right)diag\left(p{\left(\theta_{0}\right)}^{-1/2}\right) \end{array}$$

and

(10) $$W\left(\theta_{0}\right)=H\left(\theta_{0}\right){\left(B{\left(\theta_{0}\right)}^{⊤}B\left(\theta_{0}\right)\right)}^{-1}B{\left(\theta_{0}\right)}^{⊤}diag\left(p{\left(\theta_{0}\right)}^{\alpha /2}\right)diag\left(p{\left(\theta_{0}\right)}^{-1/2}\right)$$

since

$$\frac{\partial F}{\partial p(\theta_{0})}=\left(\begin{array}{c} 0_{\nu \times m}\\ -\Phi^{\prime \prime }\left(1\right)B{\left(\theta_{0}\right)}^{⊤}diag\left(p{\left(\theta_{0}\right)}^{\alpha /2}\right)diag\left(p{\left(\theta_{0}\right)}^{-1/2}\right) \end{array}\right).$$

Expanding $\theta^{*}\left(p\right)$ around $p(\theta_{0})$ and using (10) gives, for $\theta^{*}\left(p\left(\theta_{0}\right)\right)=\theta_{0}$,

$$\begin{array}{cc} \theta^{*}\left(p\right)=\; & \theta_{0}+H\left(\theta_{0}\right){\left(B{\left(\theta_{0}\right)}^{⊤}B\left(\theta_{0}\right)\right)}^{-1}B{\left(\theta_{0}\right)}^{⊤}diag\left(p{\left(\theta_{0}\right)}^{\alpha /2}\right)\times \\ & \times diag\left(p{\left(\theta_{0}\right)}^{-1/2}\right)\left(\hat{p}-p\left(\theta_{0}\right)\right)+o\left(∥\hat{p}-p\left(\theta_{0}\right)∥\right). \end{array}$$

Since $\hat{p}\overset{p}{\to }p\left(\theta_{0}\right)$ eventually $\hat{p}\in A$ and then $\gamma^{*}\left(\hat{p}\right)=\left(\lambda^{*}\left(\hat{p}\right),\theta^{*}\left(\hat{p}\right)\right)$ is the unique solution of the system

$$\begin{array}{cc} f_{k}\left(\theta \right)=0, & k=1,\ldots ,\nu \\ \frac{\partial d_{\Phi }^{\alpha }\left(p,p(\theta )\right)}{\partial \theta_{j}}+\sum_{k=1}^{\nu }\lambda_{k}\frac{\partial f_{k}\left(\theta \right)}{\partial \theta_{j}}=0, & j=1,\ldots ,s \end{array}$$

and $(\hat{p},\gamma^{*}\left(\hat{p}\right))\in U$. Hence, $\theta^{*}\left(\hat{p}\right)$ coincides with rMDE ${\hat{\theta }}_{\left(\Phi ,\alpha \right)}^{r}$ given in (9). □

The theorem below establishes the asymptotic normality of rMDE which is a straightforward extension of Theorem 2.4 [11] since by the Central Limit Theorem we know that

(11) $$\sqrt{N}\left(\hat{p}-p\left(\theta_{0}\right)\right)\overset{L}{\underset{N\to \infty }{\to }}N\left(0,\Sigma_{p(\theta_{0})}\right)$$

with the asymptotic variance-covariance matrix $\Sigma_{p(\theta_{0})}$ given by $diag\left(p\left(\theta_{0}\right)\right)-p\left(\theta_{0}\right)p{\left(\theta_{0}\right)}^{⊤}$.

**Theorem** **2.** *Under Assumptions $\left(A_{0}\right)$–$\left(A_{5}\right)$, by (11) and for $W(\theta_{0})$ given in (10), the asymptotic distribution of rMDE is the s-dimensional Normal distribution given by*

$$\sqrt{N}\left({\hat{\theta }}_{\left(\Phi ,\alpha \right)}^{r}-\theta_{0}\right)\overset{L}{\underset{N\to \infty }{\to }}N_{s}\left(0,W\left(\theta_{0}\right)\Sigma_{p(\theta_{0})}W{\left(\theta_{0}\right)}^{⊤}\right).$$

**Remark** **1.** *The proposed class of estimators forms a family of estimators that goes beyond the indicator α since it is easy to see that estimators obtained for the Csiszar’s φ family are given for α=0 in (1) and also the standard equiprobable model.*

## 3. Statistical Inference

In this section, we introduce the double index divergence test statistic

(12) $$T_{\Phi_{1}}^{\alpha_{1}}\left({\hat{\theta }}_{\left(\Phi_{2},\alpha_{2}\right)}^{r}\right)=\frac{2N}{\Phi_{1}^{\prime \prime }\left(1\right)}d_{\Phi_{1}}^{\alpha_{1}}\left(\hat{p},p({\hat{\theta }}_{\left(\Phi_{2},\alpha_{2}\right)}^{r})\right)$$

with $\Phi_{1},\Phi_{2}\in F^{*}$ and $\alpha_{1},\alpha_{2}>0$ and make the additional assumptions by which we focus on the Csiszar’s family of measures for testing purposes (the notation φ is used for clarity) and the equiprobable model:

**Assumption** **2.**

$\left(A_{6}\right)$

$\;p_{i}=1/m,\forall i$

$\left(A_{7}\right)$

*$\;\Phi_{1}=\varphi ,\alpha_{1}=0$.*

The Theorem below provides the asymptotic distribution of (12) under Assumptions $\left(A_{0}\right)$–$\left(A_{7}\right)$. Assumption $\begin{array}{c} \left(A_{7}\right) \end{array}$ will be later relaxed and a general asymptotic result will be presented in the next subsection. A discussion about Assumption $A_{6}$ will also be made in the sequel.

**Theorem** **3.** *Under Assumptions $\begin{array}{c} \left(A_{0}\right) \end{array}$–$\left(A_{7}\right)$ and for the hypothesis in (3) we have*

$$T_{\varphi }^{0}\left({\hat{\theta }}_{\left(\Phi_{2},\alpha_{2}\right)}^{r}\right)=\frac{2N}{\varphi^{\prime \prime }\left(1\right)}d_{\varphi }\left(\hat{p},p({\hat{\theta }}_{\left(\Phi_{2},\alpha_{2}\right)}^{r})\right)\overset{L}{\underset{N\to \infty }{\to }}\chi_{m-1-s-\nu }^{2}$$

*with ${\hat{\theta }}_{\left(\Phi_{2},\alpha_{2}\right)}^{r}$ given in (9).*

**Proof.**  It is straightforward that

$$p\left({\hat{\theta }}_{\left(\Phi_{2},\alpha_{2}\right)}^{r}\right)=p\left(\theta_{0}\right)+J\left(\theta_{0}\right)\left({\hat{\theta }}_{\left(\Phi_{2},\alpha_{2}\right)}^{r}-\theta_{0}\right)+o(∥{\hat{\theta }}_{\left(\Phi_{2},\alpha_{2}\right)}^{r}-\theta_{0}∥)$$

which by Theorem 2, expression (11), and for $M\left(\theta_{0}\right)=J\left(\theta_{0}\right)W\left(\theta_{0}\right)$ reduces to

$$p\left({\hat{\theta }}_{\left(\Phi_{2},\alpha_{2}\right)}^{r}\right)-p\left(\theta_{0}\right)=M\left(\theta_{0}\right)\left(\hat{p}-p\left(\theta_{0}\right)\right)+o_{p}\left(N^{-1/2}\right)$$

which implies that

(13) $$\sqrt{N}\left(p\left({\hat{\theta }}_{\left(\Phi_{2},\alpha_{2}\right)}^{r}\right)-p\left(\theta_{0}\right)\right)\overset{L}{\underset{N\to \infty }{\to }}N\left(0,M\left(\theta_{0}\right)\Sigma_{p(\theta_{0})}M{\left(\theta_{0}\right)}^{⊤}\right).$$

Combining the above we obtain

$$\sqrt{N}\left(\begin{array}{c} \hat{p}-p(\theta_{0})\\ p\left({\hat{\theta }}_{\left(\Phi_{2},\alpha_{2}\right)}^{r}\right)-p\left(\theta_{0}\right) \end{array}\right)\overset{L}{\underset{N\to \infty }{\to }}N\left(0,\left(\begin{array}{c} I\\ M(\theta_{0}) \end{array}\right)\Sigma_{p(\theta_{0})}\left(I,M{\left(\theta_{0}\right)}^{⊤}\right)\right)$$

and

$$\sqrt{N}\left(\hat{p}-p\left({\hat{\theta }}_{\left(\Phi_{2},\alpha_{2}\right)}^{r}\right)\right)\overset{L}{\underset{N\to \infty }{\to }}N\left(0,L\left(\theta_{0}\right)\right)$$

where

(14) $$L\left(\theta_{0}\right)=\Sigma_{p(\theta_{0})}-M\left(\theta_{0}\right)\Sigma_{p(\theta_{0})}-\Sigma_{p(\theta_{0})}M{\left(\theta_{0}\right)}^{⊤}+M\left(\theta_{0}\right)\Sigma_{p(\theta_{0})}M{\left(\theta_{0}\right)}^{⊤}.$$

The expansion of $d_{\varphi }\left(p,q\right)$ around $\left(p\left(\theta_{0}\right),p\left(\theta_{0}\right)\right)$ yields

$$T_{\varphi }^{0}\left({\hat{\theta }}_{\left(\Phi_{2},\alpha_{2}\right)}^{r}\right)=\sum_{i=1}^{m}\frac{N}{p_{i}\left(\theta_{0}\right)}{\left({\hat{p}}_{i}-p_{i}\left({\hat{\theta }}_{\left(\Phi_{2},\alpha_{2}\right)}^{r}\right)\right)}^{2}+o_{p}\left(1\right)=X^{⊤}X+o_{p}\left(1\right)$$

where

$$X=\sqrt{N}diag\left(p{\left(\theta_{0}\right)}^{-1/2}\right)\left(\hat{p}-p\left({\hat{\theta }}_{\left(\Phi_{2},\alpha_{2}\right)}^{r}\right)\right)\overset{L}{\underset{N\to \infty }{\to }}N\left(0,T\left(\theta_{0}\right)\right).$$

Then, under $A_{7}$, $T(\theta_{0})$ (see (14)) is a projection matrix of rank m−1−s+ν since the trace of the matrices $A\left(\theta_{0}\right){\left(A{\left(\theta_{0}\right)}^{⊤}A\left(\theta_{0}\right)\right)}^{-1}A{\left(\theta_{0}\right)}^{⊤}$ and $A\left(\theta_{0}\right){\left(A{\left(\theta_{0}\right)}^{⊤}A\left(\theta_{0}\right)\right)}^{-1}Q{\left(\theta_{0}\right)}^{⊤}$ ${\left(Q\left(\theta_{0}\right)(A{\left(\theta_{0}\right)}^{⊤}A\left(\theta_{0}\right)\right)}^{-1}Q{\left(\theta_{0}\right)}^{⊤}{)}^{-1}$ $Q(\theta_{0})$${\left(A{\left(\theta_{0}\right)}^{⊤}A\left(\theta_{0}\right)\right)}^{-1}A{\left(\theta_{0}\right)}^{⊤}$ is equal to *s* and ν, respectively.

Then, the result follows from the fact (see ([24], p. 57)) that $X^{⊤}X$ has a chi-squared distribution with degrees of freedom equal to the rank of the variance-covariance matrix of the random vector X as long as it is a projection matrix. □

**Remark** **2.** *Relaxation of Assumption $\begin{array}{c} \left(A_{6}\right) \end{array}$: Arguing as in [11], when the true model is not the equiprobable the result of Theorem 3 holds true as long as $\alpha_{2}=0$ and approximately true when $\alpha_{2}\to 0$.*

### Asymptotic Theory of the Dual Divergence Test Statistic

Having established the two main results of the work, namely the decomposition of the proposed restricted estimator (Theorem 1) together with its asymptotic properties (Theorem 2), as well as the asymptotic distribution of the associated test statistic under the class of Csiszar φ-functions (Theorem 3) we continue below extended in a natural way the results of [11] for the dual divergence test statistic. The extensions presented in this section are considered vital due to their practical impication on cross tabulations discussed in Section 4. The proofs will be omitted since both results (Theorems 4 and 5) follow along the lines of previous results (see Theorems 3.4 and 3.9 of [11]). In what follows we adopt the following notation:

$$b=m^{-\alpha_{1}},\;p_{\left(1\right)}^{\alpha_{1}}=\min_{i\in \{1,\ldots ,m\}}p_{i}{\left(\theta_{0}\right)}^{\alpha_{1}},\;p_{\left(m\right)}^{\alpha_{1}}=\max_{i\in \{1,\ldots ,m\}}p_{i}{\left(\theta_{0}\right)}^{\alpha_{1}},k=m-1-s+\nu .$$

**Theorem** **4.** *Under Assumptions $\begin{array}{c} \left(A_{0}\right) \end{array}$–$\left(A_{7}\right)$ we have*

$$T_{\Phi_{1}}^{\alpha_{1}}\left({\hat{\theta }}_{\left(\Phi_{2},\alpha_{2}\right)}^{r}\right)\overset{L}{\underset{N\to \infty }{\to }}b\chi_{k}^{2}.$$

**Remark** **3.** *Consider the case where Assumption $\begin{array}{c} \left(A_{6}\right) \end{array}$ is relaxed. Then, the asymptotic distribution of the test statistic $T_{\Phi_{1}}^{\alpha_{1}}\left({\hat{\theta }}_{\left(\Phi_{2},\alpha_{2}\right)}^{r}\right)$ is estimated to be approximately $b\chi_{k}^{2}$ where*

(15) $$b=\frac{p_{\left(1\right)}^{\alpha_{1}}+p_{\left(m\right)}^{\alpha_{1}}}{2}$$

*as long as $\alpha_{2}=0$ or $\alpha_{2}\to 0$. For further elaboration of this remark we refer to [11].*

**Remark** **4.** *Observe that if $\alpha_{1}\to 0$ then b→1 and the asymptotic distribution becomes $\chi_{k}^{2}$, while for $\alpha_{1}$ away from 0 the distribution is proportional to $\chi_{k}^{2}$ with proportionality index b≠1. However, for not equiprobable models these statements hold true as long as $\alpha_{2}$ is close to zero.*

Consider now the hypothesis with contiguous alternatives [25,26]

(16) $$H_{0}:p=p\left(\theta_{0}\right)\;vs.\;H_{1,N}:p=p\left(\theta_{0}\right)+\frac{d}{\sqrt{N}}$$

where d is an *m*-dimensional vector of known real values with components $d_{i}$ satisfying the assumption $\sum_{i=1}^{m}d_{i}=0$.

Observe that as *N* tends to infinity, the local contiguous alternative converges to the null hypothesis at the rate $O(N^{1/2})$. Alternatives, such as those in (16), are known as *Pitman transition alternatives* or *Pitman (local) alternatives* or *local contiguous alternatives* to the null hypothesis $H_{0}$ [25].

**Theorem** **5.** *Under Assumptions $\left(A_{0}\right)$–$\left(A_{7}\right)$ and for the hypothesis (16) we have*

$$T_{\Phi_{1}}^{\alpha_{1}}\left({\hat{\theta }}_{\left(\Phi_{2},\alpha_{2}\right)}^{r}\right)\overset{L}{\underset{N\to \infty }{\to }}b\chi_{k}^{2}\left(\xi^{⊤}\xi \right)$$

*which represents a non-central chi-squared distribution with k degrees of freedom and non-centrality parameter $\xi^{⊤}\xi \;$ for which ξ = $diag\left(p{\left(\theta_{0}\right)}^{-1/2}\right)\left(I-J\left(\theta_{0}\right)W\left(\theta_{0}\right)\right)d.$*

**Remark** **5.** *Observe that under Assumption $\begin{array}{c} \left(A_{6}\right) \end{array}$ ($p_{i}=1/m$) the asymptotic distribution is independent of *Φ*, $\alpha_{1}$ and $\alpha_{2}$. As a result the associated power of the test is $\mathrm{Pr}(\chi_{k}^{2}\left(\xi^{⊤}\xi \right)\geq \chi_{k,a}^{2})$ where a the 100(1−a)% percentile of the distribution. If assumption $A_{6}$ is relaxed then the distribution is approximately non-central chi-squared with proportionality index $b=\frac{p_{\left(1\right)}^{\alpha_{1}}+p_{\left(m\right)}^{\alpha_{1}}}{2}$.*

## 4. Cross Tabulations and Dual Divergence Test Statistic

In this section, we try to take advantage of the methodology proposed earlier for the analysis of cross tabulations. In particular we focus on the case of three categorical variables, say X,Y, and *Z* with corresponding, I,J, and *K*. Then, assume that the probability mass of a realization of a randomly selected subject is denoted by $p_{ijk}\left(\theta \right)=Pr\left(X=i,Y=j,Z=k\right)>0$, where here and in what follows i=1,…,I, j=1,…,J, k=1,…,K unless otherwise stated. The associated probability vector is given as $p\left(\theta \right)=\{p_{ijk}\left(\theta \right)\}$ where

$$p_{ijk}\left(\theta \right)=\left\{\begin{array}{cc} \theta_{ijk}, & \left(i,j,k)\neq (I,J,K\right)\\ 1-\underset{\left(i,j,k)\neq (I,J,K\right)}{\sum_{i=1}^{I}\sum_{j=1}^{J}\sum_{k=1}^{K}}\theta_{ijk}, & \left(i,j,k)=(I,J,K\right) \end{array}\right.$$

and the parameter space as $\Theta =\{\theta_{ijk},\left(i,j,k\right)\neq \left(I,J,K\right)\}$. The sample estimator of $p_{ijk}\left(\theta \right)$ is ${\hat{p}}_{ijk}=n_{ijk}/N$, where $n_{ijk}$ is the frequency of the corresponding (i,j,k) cell.

In this set up the dual divergence test statistics is given as

(17) $$T_{\Phi_{1}}^{\alpha_{1}}\left({\hat{\theta }}_{\left(\Phi_{2},\alpha_{2}\right)}^{r}\right)=\frac{2N}{\Phi_{1}^{\prime \prime }\left(1\right)}\sum_{i=1}^{I}\sum_{j=1}^{J}\sum_{k=1}^{K}p_{ijk}{\left({\hat{\theta }}_{\left(\Phi_{2},\alpha_{2}\right)}^{r}\right)}^{1+\alpha }\Phi_{1}\left(\frac{{\hat{p}}_{ijk}}{p_{ijk}\left({\hat{\theta }}_{\left(\Phi_{2},\alpha_{2}\right)}^{r}\right)}\right)$$

where ${\hat{p}}_{ijk}$ as above and the rMD estimator as

(18) $${\hat{\theta }}_{\left(\Phi_{2},\alpha_{2}\right)}^{r}=\arg\underset{\left\{\theta \in \Theta \subset R^{s}:\;f_{k}\left(\theta \right)=0,k=1,\ldots ,\nu \right\}}{\inf}\sum_{i=1}^{I}\sum_{j=1}^{J}\sum_{k=1}^{K}p_{ijk}{\left(\theta \right)}^{1+\alpha_{2}}\Phi_{2}\left(\frac{{\hat{p}}_{ijk}}{p_{ijk}\left(\theta \right)}\right).$$

For $\alpha_{1}$, $\alpha_{2}$=0 and special cases of the functions $\Phi_{1}$ and $\Phi_{2}$, classical restricted minimum divergence estimators and associated test statistics can be derived from (18) and (17), respectively. For example, for $\alpha_{1}$, $\alpha_{2}$=0, and $\Phi_{1}$, $\Phi_{2}$ = $\Phi_{KL}$ the likelihood ratio test statistic with the restricted maximum likelihood estimator $\left(G^{2}\left({\hat{\theta }}^{r}\right)\right)$ can be derived, while for $\Phi_{1}$, $\Phi_{2}$ = $\Phi_{\lambda }$ and λ=1 we obtain the chi-squared test statistic with the restricted minimum chi-squared estimator $\left(X^{2}\left({\hat{\theta }}_{X^{2}}^{r}\right)\right)$. For $\Phi_{1}$, $\Phi_{2}$ = $\Phi_{\lambda }$ and λ=2/3 the dual divergence test statistic reduces to the power divergence test statistic with the restricted minimum power divergence estimator $\left(CR\left({\hat{\theta }}_{CR}^{r}\right)\right)$ whereas for λ=−1/2 reduces to the Freeman–Tukey test statistic with the restricted minimum Freeman–Tukey estimator $\left(FT\left({\hat{\theta }}_{FT}^{r}\right)\right)$.

The hypothesis of conditional independence between *X*, *Y*, and *Z* is given for any triplet i,j,k by

$$H_{0}:p_{ijk}\left(\theta_{0}\right)=\frac{p_{i*k}\left(\theta_{0}\right)p_{*jk}\left(\theta_{0}\right)}{p_{**k}\left(\theta_{0}\right)},\;\theta_{0}\in \Theta \;\mathrm{unknown}$$

where

$$p_{i*k}\left(\theta_{0}\right)=\sum_{j=1}^{J}p_{ijk}\left(\theta_{0}\right),\;\;p_{*jk}\left(\theta_{0}\right)=\sum_{i=1}^{I}p_{ijk}\left(\theta_{0}\right)\;\;and\;\;p_{**k}\left(\theta_{0}\right)=\sum_{i=1}^{I}\sum_{j=1}^{J}p_{ijk}\left(\theta_{0}\right).$$

Under the (I−1)(J−1)K constrained functions

$$f_{ijk}\left(\theta \right)=p_{11k}\left(\theta \right)p_{ijk}\left(\theta \right)-p_{1jk}\left(\theta \right)p_{i1k}\left(\theta \right)=0$$

i=2,…,I,j=2,…,J,k=1,…,K the above $H_{0}$ hypothesis with $\theta_{0}$ unknown, becomes

$$H_{0}:p=p\left(\theta_{0}\right),\;\mathrm{for}\;\theta_{0}\in \Theta_{0},$$

where $\Theta_{0}=\left\{\theta \in \Theta :f_{ijk}\left(\theta \right)=0,\;i=2,\ldots ,I,\;j=2,\ldots ,J,\;k=1,\ldots ,K\right\}.$

**Remark** **6.** *For practical purposes, the choice of the values of the indices is motivated by the work of [8] where, in an attempt to achieve a compromise between robustness and efficiency of estimators, they recommended the use of small values in the (0,1) region. In the following subsection, our analysis will reconfirm their findings since as it will be seen, values of both indices close to (0) (than to one (1)) will be found to be associated with a good performance not only in terms of estimation but also in terms of goodness of fit as it will be reflected in the size and the power of the test.*

### Simulation Study

In this simulation study, we use the rMD estimator and the associated dual divergence test statistic for the analysis of cross tabulations. Specifically, we are going to compare in terms of size and power classical tests with those that can be derived through the proposed methodology, for the problem of conditional independence of three random variables in contingency tables. We test the hypothesis of conditional independence for a 2×2×2 contingency table, thus in this case we have m=8 probabilities of the multinomial model, s=7 unknown parameters to estimate and two constraint functions (ν=2) which are given by

$$f_{221}\left(\theta \right)=\theta_{111}\theta_{221}-\theta_{121}\theta_{211}\;and\;f_{222}\left(\theta \right)=\theta_{112}\left(1-\underset{\left(i,j,k)\neq (2,2,2\right)}{\sum_{i=1}^{2}\sum_{j=1}^{2}\sum_{k=1}^{2}}\theta_{ijk}\right)-\theta_{122}\theta_{212}.$$

For a better understanding of the behaviour of the dual divergence test statistic given in (17) we compare it with the four classical tests-of-fit mentioned earlier in Section 4, namely with the $G^{2}\left({\hat{\theta }}^{r}\right)$, $X^{2}\left({\hat{\theta }}_{X^{2}}^{r}\right)$, $CR({\hat{\theta }}_{CR}^{r})$ and $FT({\hat{\theta }}_{FT}^{r})$. The proposed test $T_{\Phi_{1}}^{\alpha_{1}}\left({\hat{\theta }}_{\left(\Phi_{2},\alpha_{2}\right)}^{r}\right)$ is applied for $\Phi_{1}=\Phi_{\alpha_{1}}$, $\Phi_{2}=\Phi_{\alpha_{2}}$ and six different values of $\alpha_{1}$ and $\alpha_{2}$, $\alpha_{1}$, $\alpha_{2}$ = $10^{-7}$, 0.01, 0.05, 0.10, 0.50, and 1.50. Note that, the critical values used in this simulation study, are the asymptotic critical values based on the asymptotic distribution $b\chi_{2}^{2}$ with *b* as in (15) for the double index family of test statistics, and the $\chi_{2}^{2}$ for the classical test statistics. For the analysis we used 100,000 simulations and sample sizes equal to n=20,25 (small sample sizes) and n=40,45 (moderate sample sizes).

In this study, we have used the model previously considered by [27] given by

$$\begin{array}{cccc} p_{111} & =\pi_{111}-\pi_{111}w & p_{211} & =\pi_{211}+\pi_{222}w-\pi_{111}w\\ p_{112} & =\pi_{112}+\pi_{111}w-\pi_{222}w & p_{212} & =\pi_{212}+\pi_{111}w-\pi_{222}w\\ p_{121} & =\pi_{121}+\pi_{222}w & p_{221} & =\pi_{221}+\pi_{222}w-\pi_{111}w\\ p_{122} & =\pi_{122}+\pi_{111}w & p_{222} & =\pi_{222}-\pi_{222}w \end{array}$$

where 0≤w<1 and $\pi_{ijk}=p_{i**}\times p_{*j*}\times p_{**k}$i,j,k=1,2 with

$$\begin{array}{cccccccc} \pi_{111} & =0.036254 & \pi_{112} & =0.164994 & \pi_{121} & =0.092809 & \pi_{122} & =0.133645\\ \pi_{211} & =0.092809 & \pi_{212} & =0.133645 & \pi_{221} & =0.237591 & \pi_{222} & =0.108253. \end{array}$$

For w=0 we take the model under the null hypothesis of conditional independence while for values w≠0 we take the models under the alternative hypotheses. We considered the following values of *w* = 0.00, 0.30, 0.60, and 0.90. Note that the larger the value of *w* the more we deviate from the null model. For the simulation study, we used the R software [28], while for the constrained optimization the auglag function from the nloptr package [29].

From Table 1, we can observe that in terms of size the performance of the $T_{\Phi_{1}}^{\alpha_{1}}\left({\hat{\theta }}_{\left(\Phi_{2},\alpha_{2}\right)}^{r}\right)$ is adequate for values of $\alpha_{1},\alpha_{2}$≤0.5 both for small and moderate sample sizes. In addition, we can see that for $\alpha_{1}\leq 0.10$, $T_{\Phi_{1}}^{\alpha_{1}}\left({\hat{\theta }}_{\left(\Phi_{2},\alpha_{2}\right)}^{r}\right)$ appears to be liberal while for $\alpha_{1}\geq 0.5$ appears to be conservative. We also note that the size becomes smaller as $\alpha_{1}$ and $\alpha_{2}$ increase with $\alpha_{1}\geq \alpha_{2}$. Table 2 provides the size of the classical tests-of-fit from where we can observe that $CR({\hat{\theta }}_{CR}^{r})$ has the best performance among all competing tests for every sample size. In contrast, $FT({\hat{\theta }}_{FT}^{r})$ has the worst performance among all competing tests and appears to be very liberal. Furthermore, $X^{2}\left({\hat{\theta }}_{X^{2}}^{r}\right)$ appears to be conservative while $G^{2}\left({\hat{\theta }}^{r}\right)$ appears to be liberal. Note that for $\alpha_{1}\in \left[0.01,0.5\right]$ and $\alpha_{2}\leq 0.10$, $T_{\Phi_{1}}^{\alpha_{1}}\left({\hat{\theta }}_{\left(\Phi_{2},\alpha_{2}\right)}^{r}\right)$ behaves better than the $G^{2}\left({\hat{\theta }}^{r}\right)$ test statistic and its performance is quite close to the performance of the $X^{2}\left({\hat{\theta }}_{X^{2}}^{r}\right)$.

In order to examine the closeness of the estimated (true) size to the nominal size α=0.05 we consider the criterion given by Dale [30]. The criterion involves the following inequality

(19) $$|\mathrm{logit}\left(1-{\hat{\alpha }}_{n}\right)-\mathrm{logit}\left(1-\alpha \right)|\leq d$$

where logit(p)=log(p/(1−p)) and ${\hat{\alpha }}_{n}$ is the estimated (true) size. The estimated (true) size is considered to be close to the nominal size if (19) is satisfied with d=0.35. Note that in this situation the estimated (true) size is close to the nominal one if ${\hat{\alpha }}_{n}\in \left[0.0357,0.0695\right]$ and is presented in Table 1 and Table 2 in bold. This criterion has been used previously among others by [27,31].

Regarding the proposed test we can see that for small sample sizes the estimated (true) size is close to the nominal for $\alpha_{1}\in \left[0.10,0.50\right]$ and $\alpha_{2}\leq 0.10$ while for moderate sample sizes for $\alpha_{1}\in \left[10^{-7},0.50\right]$ and $\alpha_{2}\leq 0.10$. With reference to the classical tests-of-fit we can observe that the size of the $CR({\hat{\theta }}_{CR}^{r})$ is close to the nominal for every sample size whereas the size of $G^{2}\left({\hat{\theta }}^{r}\right)$ and $X^{2}\left({\hat{\theta }}_{X^{2}}^{r}\right)$ is close only for moderate sample sizes. Finally, we note that the estimated (true) size of $FT({\hat{\theta }}_{FT}^{r})$ fails to be close to the nominal both for small and moderate sample sizes.

In Table 3, Table 4 and Table 5, we provide the results regarding the power of the proposed family of test statistics for the three alternatives and sample sizes n=20,25,40,45, while Table 2 provides the results regarding the power of the classical tests-of-fit. The performance tends to be better as we deviate from the null model and as the sample size increases both for the classical and the proposed tests.

As general comments regarding the behaviour of the proposed and the classical tests-of-fit in terms of power we state that the best results for the $T_{\Phi_{1}}^{\alpha_{1}}\left({\hat{\theta }}_{\left(\Phi_{2},\alpha_{2}\right)}^{r}\right)$ are obtained for small values of $\alpha_{1}$ in the range (0,0.1] and large values of $\alpha_{2}$ with $\alpha_{1}\leq \alpha_{2}$. Note that although in terms of power results become better as $\alpha_{2}$ increases in terms of size these are adequate only for $\alpha_{2}\leq 0.5$. In addition, we can observe that the performance of $T_{\Phi_{1}}^{\alpha_{1}}\left({\hat{\theta }}_{\left(\Phi_{2},\alpha_{2}\right)}^{r}\right)$ is better than the $CR({\hat{\theta }}_{CR}^{r})$ and $X^{2}\left({\hat{\theta }}_{X^{2}}^{r}\right)$ for every alternative and every sample size for $\alpha_{1}\leq 0.1$ and $\alpha_{2}\leq 0.5$ and slightly better than $G^{2}\left({\hat{\theta }}^{r}\right)$ for small values of $\alpha_{1}$ and large values of $\alpha_{2}$, for example for $\alpha_{1}=0.01$ and $\alpha_{2}=0.50$. Furthermore, we can observe that for $\alpha_{1}=0.1$ and $\alpha_{2}\leq 0.1$ the size of the test is better than the size of the $G^{2}\left({\hat{\theta }}^{r}\right)$ and slightly worst form the size of the $CR({\hat{\theta }}_{CR}^{r})$ and $X^{2}\left({\hat{\theta }}_{X^{2}}^{r}\right)$ test statistics while its power is quite better than the power of the $CR({\hat{\theta }}_{CR}^{r})$ and $X^{2}\left({\hat{\theta }}_{X^{2}}^{r}\right)$ and slightly worst than the $G^{2}\left({\hat{\theta }}^{r}\right)$. Additionally, we can see that as $\alpha_{1}$ and $\alpha_{2}$ tend to 0 the behaviour of the $T_{\Phi_{1}}^{\alpha_{1}}\left({\hat{\theta }}_{\left(\Phi_{2},\alpha_{2}\right)}^{r}\right)$ test statistic coincides with the $G^{2}\left({\hat{\theta }}^{r}\right)$ test both in terms of size and power as it was expected.

**Figure 1:**
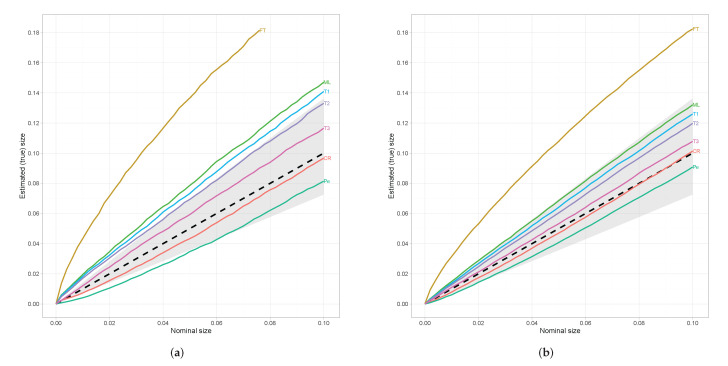
Estimated (true) sizes against nominal sizes. The shaded area refers to Dale’s criterion. (**a**) n=25. (**b**) n=45.

In order to attain a better insight about the behaviour of the test statistics, we apply Dale’s criterion, not only for the nominal size α=0.05, but also for a range of nominal sizes that are of interest. Based on the previous analysis, beside the classical tests, we will focus our interest on the $T_{\Phi_{1}}^{0.05}\left({\hat{\theta }}_{\left(\Phi_{2},0.05\right)}^{r}\right)$, $T_{\Phi_{1}}^{0.10}\left({\hat{\theta }}_{\left(\Phi_{2},0.10\right)}^{r}\right)$, and $T_{\Phi_{1}}^{0.20}\left({\hat{\theta }}_{\left(\Phi_{2},0.20\right)}^{r}\right)$. The following simplified notation is used in every Figure, FT≡$FT({\hat{\theta }}_{FT}^{r})$, ML≡$G^{2}\left({\hat{\theta }}^{r}\right)$, CR≡$CR({\hat{\theta }}_{CR}^{r})$, Pe≡$X^{2}\left({\hat{\theta }}_{X^{2}}^{r}\right)$, T1≡$T_{\Phi_{1}}^{0.05}\left({\hat{\theta }}_{\left(\Phi_{2},0.05\right)}^{r}\right)$, T2≡$T_{\Phi_{1}}^{0.10}\left({\hat{\theta }}_{\left(\Phi_{2},0.10\right)}^{r}\right)$, and T3 = $T_{\Phi_{1}}^{0.20}\left({\hat{\theta }}_{\left(\Phi_{2},0.20\right)}^{r}\right)$. From Figure 1a, we can see that for small sample sizes (n=25)$T_{\Phi_{1}}^{0.20}\left({\hat{\theta }}_{\left(\Phi_{2},0.20\right)}^{r}\right)$ and $CR({\hat{\theta }}_{CR}^{r})$ satisfy Dale’s criterion for every nominal size while $T_{\Phi_{1}}^{0.10}\left({\hat{\theta }}_{\left(\Phi_{2},0.10\right)}^{r}\right)$ and $X^{2}\left({\hat{\theta }}_{X^{2}}^{r}\right)$ for nominal sizes greater than 0.03 and 0.06, respectively. Note that the dashed line in Figure 1 denotes the situation in which the estimated (true) size equals to the nominal size and thus lines that lie above this reference line refer to liberal tests while those that lie below to conservative ones. On the other hand, for moderate sample sizes (n=45) all chosen test statistics satisfy Dale’s criterion except $FT({\hat{\theta }}_{FT}^{r})$.

Taking into account the fact that the actual size of each test differs from the targeted nominal size, we have to make an adjustment in order to proceed further with the comparison of the tests in terms of power. We focus our interest in those tests that satisfy Dale’s criterion and follow the method proposed in [32] which involves the so-called receiver operating characteristic (ROC) curves. In particular, let G(t)=Pr(T≥t) be the survivor function of a general test statistic *T*, and c=inf{t:G(t)≤α} be the critical value, then ROC curves can be formulated by plotting the power $G_{1}\left(c\right)$ against the size $G_{0}\left(c\right)$ for various values of the critical value *c*. Note that with $G_{0}\left(t\right)$ we denote the distribution of the test statistic under the null hypothesis and with $G_{1}\left(t\right)$ under the alternative.

**Figure 2:**
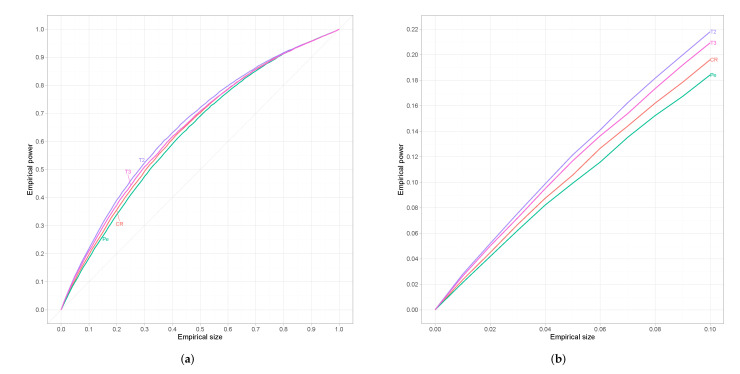
(**a**) Empirical ROC curves for *n* = 25. (**b**) The same curves magnified over a relevant range of empirical sizes.

**Figure 3:**
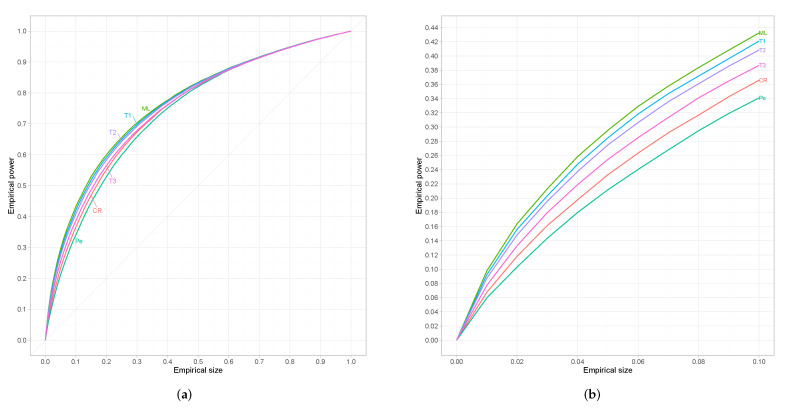
(**a**) Empirical ROC curves for *n* = 45. (**b**) The same curves magnified over a relevant range of empirical sizes.

Since results are similar for every alternative we restrict ourselves to w=0.60 which refers to an alternative that is neither too close nor too far from the null. For small sample sizes (n=25) results are presented in Figure 2, where we can see that the proposed test is superior from the classical tests-of-fit in terms of power. However, for moderate sample sizes (n=45) we can observe in Figure 3 that $G^{2}\left({\hat{\theta }}^{r}\right)$ has the best performance among all competing tests followed by the proposed test-of-fit.

From the conducted analysis we conclude that regarding the proposed test there is a trade off between size and power for different choices of the indices $\alpha_{1}$ and $\alpha_{2}$. In particular, we can see that as $\alpha_{1}$ increases the size becomes smaller in the expense of smaller power, while as $\alpha_{2}$ increases the power becomes better and the tests more liberal. In conclusion, we could state that for values of $\alpha_{1}$ and $\alpha_{2}$ in the range (0.05,0.25) the resulting test statistic provides a fair balance between size and power which makes it an attractive alternative to the classical tests-of-fit where for small sample sizes larger values of the indices are preferable whereas for moderate sample sizes, smaller ones are recommended.

## 5. Conclusions

In this work, a general divergence family of test statistics is presented for hypothesis testing problems as in (3), under constraints. For estimating purposes, we introduce, discuss and use the rMD (restricted minimum divergence) estimator presented in (8). The proposed double index (dual) divergence test statistic involves two pairs of elements, namely $\left(\Phi_{2},\alpha_{2}\right)$ to be used for the estimation problem and $\left(\Phi_{1},\alpha_{1}\right)$ to be used for the testing problem. The duality refers to the fact that the two pairs may or may not be the same providing the researcher with the greatest possible flexibility.

The asymptotic distribution of the dual divergence test statistic is found to be proportional to the chi-squared distribution irrespectively of the nature of the multinomial model, as long as the values of the two indicators involved are relative close to zero (less than 0.5). Such values are known to provide a satisfactory balance between efficiency and robustness (see, for instance, [8] or [3]).

The methodology developed in this work can be used in the analysis of contingency tables which is applicable in various scientific fields: biosciences, such as genetics [33] and epidemiology [34]; finance, such as the evaluation of investment effectiveness or business performance [35]; insurance science [36]; or socioeconomics [37]. This work concludes with a comparative simulation study between classical test statistics and members of the proposed family, where the focus is placed on the conditional independence of three random variables. Results indicate that, by selecting wisely the values of the $\alpha_{1}$ and $\alpha_{2}$ indices, we can derive a test statistic that can be thought of as a powerful and reliable alternative to the classical tests-of-fit especially for small sample sizes.

## Figures and Tables

**Table 1:** Size (w=0.00) calculations (%) of the $T_{\Phi_{1}}^{\alpha_{1}}\left({\hat{\theta }}_{\left(\Phi_{2},\alpha_{2}\right)}^{r}\right)$ test statistic for sample sizes n=20,25,40,45. Sizes that satisfy Dale’s criterion are presented in bold.

	$\alpha_{2}$											
$\alpha_{1}$	$10^{-7}$	0.01	0.05	0.10	0.50	1.50	$10^{-7}$	0.01	0.05	0.10	0.50	1.50
	n=20						n=25					
$10^{-7}$	8.256	8.257	8.260	8.263	9.216	13.856	7.863	7.865	7.878	7.920	8.927	13.192
0.01	8.207	8.206	8.209	8.224	9.224	13.623	7.753	7.754	7.763	7.817	8.797	12.930
0.05	7.896	7.849	7.879	7.886	8.719	12.916	7.340	7.334	7.327	7.350	8.313	12.277
0.10	7.403	7.404	7.378	7.356	8.046	11.994	**6.965**	**6.959**	**6.940**	**6.934**	7.675	11.364
0.50	**3.873**	**3.850**	**3.769**	**3.612**	3.023	4.050	**3.857**	**3.819**	**3.722**	**3.604**	3.191	4.304
1.50	0.920	0.893	0.807	0.758	0.509	0.202	1.046	1.019	0.948	0.885	0.602	0.203
	n=40						n=45					
$10^{-7}$	7.016	7.016	7.027	7.055	7.887	11.362	**6.858**	**6.858**	**6.870**	**6.908**	7.732	11.099
0.01	6.933	6.933	6.940	6.957	7.778	11.183	**6.760**	**6.760**	**6.770**	**6.805**	7.601	10.941
0.05	**6.590**	**6.589**	**6.580**	**6.593**	7.342	10.505	**6.427**	**6.422**	**6.415**	**6.426**	7.153	10.340
0.10	**6.246**	**6.239**	**6.228**	**6.222**	6.794	9.758	**6.082**	**6.070**	**6.053**	**6.043**	6.612	9.586
0.50	**3.854**	**3.832**	**3.762**	**3.661**	3.367	4.362	**3.813**	**3.789**	**3.716**	**3.635**	3.331	4.269
1.50	1.172	1.160	1.115	1.066	0.760	0.383	1.183	1.170	1.119	1.068	0.773	0.437

**Table 2:** Size (w=0.00) and power (w=0.30,0.60,0.90) calculations (%) for the classical tests-of-fit. Sizes that satisfy Dale’s criterion are presented in bold.

Sample size	FT	$G^{2}$	CR	$X^{2}$	FT	$G^{2}$	CR	$X^{2}$
	w=0.00				w=0.30			
n=20	14.715	8.261	**4.219**	3.140	18.366	9.072	4.200	2.966
n=25	13.664	7.865	**4.333**	3.477	19.674	9.846	4.783	3.646
n=40	11.154	7.016	**4.722**	**4.059**	21.920	12.192	6.935	5.548
n=45	10.787	**6.858**	**4.703**	**4.082**	22.467	12.992	7.471	6.081
	w=0.40				w=0.45			
n=20	29.707	14.936	7.096	4.910	47.859	26.721	13.789	9.704
n=25	35.768	18.966	9.469	7.118	62.810	38.023	20.147	15.296
n=40	48.366	31.513	18.780	15.030	85.773	69.599	47.644	39.481
n=45	50.821	35.381	22.367	18.217	89.108	76.685	57.000	48.451

**Table 3:** Power (w=0.30) calculations (%) of the $T_{\Phi_{1}}^{\alpha_{1}}\left({\hat{\theta }}_{\left(\Phi_{2},\alpha_{2}\right)}^{r}\right)$ test statistic for sample sizes n=20,25,40,45.

	$\alpha_{2}$											
$\alpha_{1}$	$10^{-7}$	0.01	0.05	0.10	0.50	1.50	$10^{-7}$	0.01	0.05	0.10	0.50	1.50
	n=20						n=25					
$10^{-7}$	9.073	9.072	9.071	9.076	9.993	15.062	9.846	9.846	9.868	9.895	10.924	15.729
0.01	8.990	8.989	8.988	9.006	9.948	14.724	9.630	9.630	9.651	9.727	10.712	15.343
0.05	8.350	8.278	8.340	8.357	9.231	13.819	9.033	9.008	8.990	9.022	9.876	14.332
0.10	7.694	7.696	7.626	7.616	8.273	12.656	8.225	8.216	8.194	8.188	8.890	13.111
0.50	3.751	3.717	3.607	3.418	2.889	4.199	3.797	3.761	3.656	3.581	3.252	4.620
1.50	0.793	0.764	0.676	0.630	0.415	0.163	0.820	0.810	0.756	0.718	0.479	0.158
	n=40						n=45					
$10^{-7}$	12.192	12.193	12.207	12.231	13.142	17.775	12.992	12.992	13.003	13.052	14.014	18.490
0.01	11.935	11.934	11.942	11.979	12.853	17.387	12.724	12.724	12.730	12.764	13.721	18.148
0.05	11.075	11.075	11.069	11.074	11.844	16.046	11.799	11.786	11.760	11.768	12.628	16.815
0.10	10.072	10.060	10.039	10.022	10.565	14.549	10.747	10.729	10.688	10.669	11.218	15.183
0.50	4.863	4.842	4.743	4.648	4.342	5.815	5.214	5.179	5.078	4.977	4.648	6.116
1.50	0.979	0.970	0.928	0.890	0.662	0.379	1.032	1.019	0.978	0.928	0.693	0.412

**Table 4:** Power (w=0.60) calculations (%) of the $T_{\Phi_{1}}^{\alpha_{1}}\left({\hat{\theta }}_{\left(\Phi_{2},\alpha_{2}\right)}^{r}\right)$ test statistic for sample sizes n=20,25,40,45.

	$\alpha_{2}$											
$\alpha_{1}$	$10^{-7}$	0.01	0.05	0.10	0.50	1.50	$10^{-7}$	0.01	0.05	0.10	0.50	1.50
	n=20						n=25					
$10^{-7}$	14.928	14.937	14.932	14.944	16.186	22.900	18.965	18.964	19.004	19.042	20.607	27.684
0.01	14.807	14.813	14.808	14.833	16.117	22.486	18.565	18.564	18.598	18.702	20.235	27.069
0.05	13.711	13.583	13.726	13.735	14.939	21.143	17.436	17.383	17.360	17.422	18.733	25.365
0.10	12.612	12.619	12.529	12.525	13.217	19.545	15.794	15.767	15.743	15.726	16.869	23.368
0.50	6.088	5.994	5.811	5.416	4.553	6.403	6.879	6.821	6.656	6.473	5.912	8.458
1.50	1.118	1.077	0.944	0.889	0.553	0.215	1.275	1.240	1.152	1.081	0.729	0.260
	n=40						n=45					
$10^{-7}$	31.513	31.518	31.533	31.608	33.469	40.799	35.381	35.381	35.404	35.465	37.411	44.556
0.01	30.904	30.903	30.925	30.999	32.868	40.221	34.848	34.845	34.863	34.941	36.744	43.942
0.05	28.949	28.946	28.938	28.956	30.509	37.756	32.727	32.716	32.697	32.715	34.310	41.510
0.10	26.504	26.485	26.434	26.398	27.631	34.747	30.146	30.110	30.051	30.014	31.289	38.456
0.50	11.949	11.867	11.598	11.409	10.830	14.703	14.052	13.966	13.632	13.321	12.731	16.901
1.50	1.797	1.761	1.692	1.578	1.142	0.716	1.973	1.945	1.870	1.776	1.295	0.838

**Table 5:** Power (w=0.90) calculations (%) of the $T_{\Phi_{1}}^{\alpha_{1}}\left({\hat{\theta }}_{\left(\Phi_{2},\alpha_{2}\right)}^{r}\right)$ test statistic for sample sizes n=20,25,40,45.

	$\alpha_{2}$											
$\alpha_{1}$	$10^{-7}$	0.01	0.05	0.10	0.50	1.50	$10^{-7}$	0.01	0.05	0.10	0.50	1.50
	n=20						n=25					
$10^{-7}$	26.712	26.710	26.707	26.711	28.495	37.924	38.017	38.016	38.132	38.191	40.982	50.954
0.01	26.589	26.586	26.585	26.613	28.718	37.421	37.365	37.364	37.456	37.645	40.482	50.206
0.05	25.437	25.267	25.531	25.502	27.170	35.979	35.674	35.559	35.526	35.643	38.260	48.187
0.10	24.287	24.284	24.232	24.172	24.868	33.946	33.014	32.939	32.867	32.854	35.184	45.569
0.50	12.003	11.780	11.424	10.772	8.807	11.665	14.353	14.226	13.870	13.560	12.312	16.886
1.50	1.731	1.662	1.489	1.422	0.904	0.298	2.268	2.226	2.026	1.916	1.387	0.506
	n=40						n=45					
$10^{-7}$	69.599	69.605	69.637	69.755	72.196	79.363	76.685	76.685	76.731	76.805	78.802	84.683
0.01	68.923	68.923	68.954	69.049	71.518	79.003	76.177	76.173	76.192	76.264	78.143	84.344
0.05	66.310	66.309	66.306	66.365	68.576	77.069	73.760	73.745	73.732	73.766	75.748	82.751
0.10	62.500	62.455	62.372	62.343	64.660	74.161	70.295	70.264	70.144	70.131	72.172	80.319
0.50	30.094	29.904	29.349	28.848	27.895	36.902	36.612	36.465	35.792	35.073	34.056	43.732
1.50	3.748	3.678	3.472	3.210	2.269	1.562	4.349	4.274	4.017	3.747	2.665	1.927

## Data Availability

Not applicable.

## Appendix A

The Birch regularity conditions mentioned in Assumption (A5) of Section 2 are stated below (for details see [22])

1. The point $\theta_{0}$ is an interior point of Θ;
2. $p_{i}=p_{i}\left(\theta_{0}\right)>0$ for i=1,…,m;
3. The mapping p(θ):Θ→P is totally differentiable at $\theta_{0}$ so that the partial derivatives of $p_{i}\left(\theta_{0}\right)$ with respect to each $\theta_{j}$ exist at $\theta_{0}$ and p(θ) has a linear approximation at $\theta_{0}$ given by
  $$p_{i}\left(\theta \right)=p_{i}\left(\theta_{0}\right)+\sum_{j=1}^{s}\left(\theta_{j}-\theta_{0j}\right)\frac{\partial p_{i}\left(\theta_{0}\right)}{\partial \theta_{j}}+o\left(∥\theta -\theta_{0}∥\right),\;i=1,\ldots ,m$$
  as $\theta \to \theta_{0}$.
4. The Jacobian matrix
  $$J\left(\theta_{0}\right)={\left(\frac{\partial p(\theta )}{\partial \theta }\right)}_{\theta =\theta_{0}}={\left(\frac{\partial p_{i}\left(\theta_{0}\right)}{\partial \theta_{j}}\right)}_{\begin{array}{c} i=1,\ldots ,m\\ j=1,\ldots ,s \end{array}}$$
  is of full rank;
5. The mapping inverse to θ→p(θ) exists and is continuous at $\theta_{0}$;
6. The mapping p:Θ→P is continuous at every point θ∈Θ.
